# Supplementary material for: Announcing the Genome Atlas of Bamboo and Rattan (GABR) project: promoting research in evolution and in economically and ecologically beneficial plants
Source: Gigascience. 2017 Jun 16;6(7):1–7. doi: 10.1093/gigascience/gix046 (PMC5570132; doi:10.1093/gigascience/gix046)
Supplement: GIGA-D-17-00120_Original-Submission.pdf [file gix046_GIGA-D-17-00120_Original-Submission.pdf]

Manuscript Click here to download Manuscript commentary.docx

Commentary

Announcing the GABR (Genome Atlas of Bamboo and Rattan) Project: Promoting Research in Evolution and in Economically and Ecologically Beneficial Plants

Hansheng Zhao[1†], Shancen Zhao[2†], International Network for Bamboo and Rattan[3†], Benhua Fei[1], Huan Liu[2], Huanming Yang[2], Honghai Dai[1], Dan Wang[1], Wei Jin[3], Feng Tang[1], Qiang Gao[2], Hang Xun[1], Yuwei Wang[1], Lianghua Qi[1,4,5], Xianghua Yue[1,6], Shuyan Lin[7], Lianfeng Gu[8], Lubin Li[9], Tiansheng Zhu[10], Qiang Wei[11,12], Zhen Su[13], Tarmeze Bin Wan Ariffin Wan[14], Daniel A. Ofori[15], George Mbeva Muthike[16], Yigardu Mulatu Mengesha[17], Roberto Magno de Castro e Silva[18], Antonio Ludovico Beraldo[19], Zhimin Gao[1*], Xin Liu[2*], and Zehui Jiang[1*]

* Correspondence: gaozhimin@icbr.ac.cn; liuxin@genomics.cn; jiangzehui@icbr.ac.cn

† Equal contributors

1. State Forestry Administration Key Open Laboratory on the Science and Technology of Bamboo and Rattan, International Center for Bamboo and Rattan, Beijing 100102, China
2. BGI-Shenzhen, Shenzhen 518083, China
3. International Network for Bamboo and Rattan, Beijing 100102, China
4. Hainan Sanya National Positioning and Monitoring Station for Ecosystem of Bamboo and Rattan Associated Forest, Sanya 572000, China
5. Research Institute of Tropical Forest Plants, ICBR, China
6. Anhui Taiping Experimental Staition ICBR, Huangshan Anhui PRC, China
7. Nanjing Forestry University, Bamboo Research Institute, Nanjing Jiangsu 210037, China
8. Basic Forestry and Proteomics Research Center, Haixia Institute of Science and Technology, Fujian Agriculture and Forestry University, Fuzhou 350002, China
9. Research Institute of Forestry, Chinese Academy of Forestry, Beijing 100091, China
10. School of Computer Science, Fudan University, Shanghai 200433, China
11. Co-Innovation Center for Sustainable Forestry in Southern China, Nanjing Forestry University,

Nanjing, Jiangsu 210037, China

12. Bamboo Research Institute, Nanjing Forestry University, Nanjing, Jiangsu 210037, China;
13. State Key Laboratory of Plant Physiology and Biochemistry, College of Biological Sciences, China Agricultural University, Beijing 100193, China
14. FOREST PROD DIV., Forest Research Institute Malaysia (FRIM), Kepong Selangor 52109, Malaysia
15. Forestry Research Institute of Ghana P. O. Box UP 63 KNUST Kumasi, Ghana
16. Kenya Forestry Research Institute P.O. Box 20412 - 00200 Nairobi, Kenya
17. Ethiopian Environment and Forest Research Institute (EEFRI), Addis Ababa, Ethiopia
18. Federal University of Goiás (UFG), Goiania, Goiás, Brazil
19. School of Agricultural Engineering, University of Campinas, Campinas, Brazil

Email:

Hansheng Zhao: zhaohansheng@icbr.ac.cn

Shancen Zhao: zhaoshancen@genomics.cn

INBAR: zyli@inbar.int

Benhua Fei: feibenhua@icbr.ac.cn

Huan Liu: liuhuan@genomics.cn

Huanming Yang: yanghuanming@genomics.cn

Honghai Dai: h_hdai@icbr.ac.cn

Dan Wang: wangdan@icbr.ac.cn

Wei Jin: wjin@inbar.int

Feng Tang: tangfeng@icbr.ac.cn

Qiang Gao: gaoqiang@genomics.cn

Hang Xun: xunhang@icbr.ac.cn

Yuwei Wang: wangyuwei@icbr.ac.cn

Lianghua Qi: qlh@icbr.ac.cn

Xianghua Yue: yuexianghua@icbr.ac.cn

Shuyan Lin: 690952866@qq.com

Lianfeng Gu: lfgu@fafu.edu.cn

Lubin Li: lilubin@126.com

Tiansheng Zhu: tszhu@fudan.edu.cn

Qiang Wei: weiqiang@njfu.edu.cn

Zhen Su: zhensu@cau.edu.cn

Tarmeze Bin Wan Ariffin Wan: drwantarmeze@gmail.com

Daniel A. Ofori: dodori12@gmail.com

George Mbeva Muthike: muthikegm@yahoo.com

Yigardu Mulatu Mengesha: yigardumulatu@gmail.com

Roberto Magno de Castro e Silva: roberto@embambu.com.br

Antonio Ludovico Beraldo: beraldo@gmail.com

Zhimin Gao: gaozhimin@icbr.ac.cn

Xin Liu: liuxin@genomics.cn

Zehui Jiang: jiangzehui@icbr.ac.cn

Abstract

Bamboo and rattan (BR) represent several plant genera and species that have long been widely grown as raw materials for manufacturing, as horticultural species for landscaping and as agroforestry species for reforestation. Today, many BR-producing countries can also harness these resources to help reduce poverty and boost regional economic growth. Growing BR could also help mitigate climate change and protect the natural environment as an essential part of sustainable development plans. However, despite much progress in research on BR, we still lack sufficient molecular and genomic resources to study these useful species. Therefore, we have launched an international collaboration, the GABR (the Genome Atlas of Bamboo and Rattan) Project, as a comprehensive and coordinated effort to accelerate our understanding of the genetics of BR through the application of genome analysis technologies. GABR includes two core subprojects: Bamboo-T1K (Transcriptomes of 1000 bamboos) and Rattan-G5 (Genomes of 5 rattans), as well as having several other cooperative subprojects. Here we describe the GABR organization and project directions and status.

Keywords: GABR, Bamboo, Rattan, Large-scale, Multi-omics, Biodiversity

Background

Bamboo species belong to the subfamily Bambusoideae in the grass family Poaceae, with substantial phenotypical diversities (**Figure 1**). Approximately 1,250 bamboo species in 75 genera have been reported. They occupy a broad range of environments around the world, from tropical and warm temperate ecosystems to cold temperate regions [1] (**Figure 2A**). Rattans are a type of spiny climbing palm that belong to the subfamily Calamoideae in the palm family Arecaceae and are native to the tropical and subtropical regions in the Eastern Hemisphere. There are ~600 rattan species in 13 genera, among which more than half are distributed in Asia with the remaining species found in West Africa and on islands in the northern Pacific [1] (**Figure 2B**). Bamboos and rattans can be used for different purposes (**Additional file 1**), including food, energy production, fiber, building materials and utensils. Thus, they have made enormous contributions to human civilization and have great potential for poverty reduction, industrial development and sustainable development.

Today, plant-derived natural resources, particularly wood resources, are threatened by environmental change because trees have naturally long growth cycles. Unlike trees, BR can be harvested as perennial crops every 3–5 years for bamboo and every 5–7 years for rattan after initial planting without causing deforestation or resource loss. BR species grow new shoots each year after the root system has become completely established underground. Moreover, due to their special fiber and wood characteristics, BR species are used industrially on a large scale. International Network for Bamboo and Rattan (INBAR) estimates that global trade in BR amounted to about $60 billion in 2015 and has increased by ~$2.5 billion annually [2]. There are about 1.5 billion people in the world whose lives are significantly associated with the use or production of BR resources [2].

Molecular genetic research has become extremely important to promote genetic, evolutionary, taxonomic, and functional studies of BR to comprehensively understand the biology and other characteristics of these genera, and to rationally utilize BR resources. Globally, the International Centre for Bamboo and Rattan (ICBR) plays a significant role globally in BR research and development. As one of the main organizations involved in BR research, the ICBR has participated in the Bamboo Genome Project (BGP) and released the draft genome of Moso bamboo (*Phyllostachys edulis*) [3] in February 2013. This species is the only whole genome sequence that is

publicly available. Aside from this, other genomic resources for BR remain limited, with a focus on a small fraction of extant BR species, providing a total of 53 transcriptomes from two bamboos and 8 transcriptomes of a single rattan species publicly available in National Center for Biotechnology Information (NCBI) Sequence Read Archive (SRA) database (**Additional file 2**).

The lack of more extensive genomic resources seriously impedes progress in BR classification and evolutionary, and functional analyses of BR. We have, therefore, launched the GABR Project, with the aim to generate large-scale ‘omics data at multiple levels to propel BR studies ranging from basic molecular biology research to applied genetic engineering.

The GABR Consortium

The GABR Consortium was established in 2016 by INBAR, an intergovernmental organization that was founded in 1997 through a treaty deposited with the United Nations. INBAR is made up of members from the many countries that have major bamboo and rattan resources, and has a professional team composed of experts in bamboo and rattan, forestry, natural resource management, ecosystem services, socio-economics, capacity building and knowledge sharing. INBAR initiated the GABR Consortium as an international, collaborative and non-profit initiative to generate comprehensive BR genomes and other ‘omics datasets to improve the conservation and utilization of the world’s BR resources. The consortium now consists of ~100 scientists from 52 scientific institutions and universities across 42 countries and one intergovernmental organization (**Additional file 3**). The Steering Committee is composed of two core consortium members who are recognized experts in taxonomy, bioinformatics, phylogeny and evolution. The head office of the GABR Consortium is located within the ICBR in Beijing, China. In addition, the GABR Consortium has chosen BGI (formerly known as Beijing Genomics Institute) in Shenzhen, China as a hub to facilitate sample collection and data sharing. INBAR, as the major member and initiator of the GABR Project, will provide support for this project.

As an international collaboration, the GABR Consortium invites any experts and institutions from related fields in the world to participate in the project. Interested participants should i) provide

additional BR resources not included in the current sample list (**Table 1**); ii) contribute to data generation or iii) address further questions in BR using GABR ‘omics data. Researchers interested in participating in the GABR Project are required to provide a brief proposal *via* email to Professor Zhimin Gao at GABR@icbr.ac.cn. Submitted proposals should include intended contributions to the GABR Project and a detailed clearly described research plan. The GABR Consortium will review submitted proposals and if appropriate substantial contributions to the project are proposed, the GABR Consortium may agree to accept the applicant as a new member of the GABR Project. Otherwise, reasons for rejection and suggestions for improvement of applications will be provided.

Project development and current progress

The GABR project includes two core subprojects, Bamboo-T1K (Transcriptomes of 1,000 bamboos) and Rattan-G5 (Genomes of 5 rattans), and we included several studies of critical pathways of BR using high-throughput sequencing data. The first phase of the GABR project has three main objectives.

First, we selected ~ 220 representative Old World woody bamboo species belonging to 37 genera, based on morphological characteristics, to use in well-resolved nuclear phylogenomic analyses to reveal the phylogenetic relationships among bamboo genera (**Item 1** in **Table 2**). We then chose ~ 340 bamboo genera for classification studies using transcriptomic sequencing and DNA barcode analysis technologies (**Item 2**).

Second, we chose two rattan species (*Daemonorops jenkinsiana* and *Calamus simplicifolius*) of economic significance to perform whole-genome sequencing and genome assembly using Illumina and PacBio platforms (**Item 3**).

Third, as we obtain data, we plan to use large-scale multi-omics data to explore critical biological phenotypes of bamboo, including the bamboo rapid-growth trait from different perspectives (**Items 4–6**), flowering development (**Item 7**) and the regulation of important metabolites using proteomics and metabolomics technologies (**Items 8** and **9**). The large-scale multi-omics data from this project will also be systematically and comprehensively analyzed using in-depth data-mining methods

(**Item 10**). For this work, we will develop novel bioinformatics methods or pipelines for the assembly and annotation of large genomes and for multi-omics analyses in plants (**Item 11**).

Currently GABR Project has started the sampling and data generation. The GABR team has established a collaborative global network to collected ~340 bamboo and two rattan samples from Malaysia, Ghana, Kenya, Ethiopia, Brazil, and many locations in China (**Additional file 4**). For the two rattan samples, flow cytometry analysis and whole genome survey have been conducted to estimate the genome size (**Additional file 5**). For the other samples, members of the project are currently carrying out DNA and RNA extraction, as well as data generation.

Data sharing policies of GABR

For data sharing, we will be following the Fort Lauderdale rules [4] and Toronto International Data Release Workshop guidelines [5]. Initially, the data will be shared at the official GABR website (www.gabr-project.com), formerly BambooGDB (bamboo genome database) [6]. All of the raw sequence data generated by the GABR Project will to be uploaded and deposited at NCBI (https://www.ncbi.nlm.nih.gov/sra) as well as to the GABR Project database. Data deposition will be released at the time of publication of any resulting papers if not sooner.

To effectively expand our understanding of BR genomes and facilitate future studies related to the GABR Project, we will also develop a GABR website where all available public BR data will be aggregated (both from the GABR Project and from previous BR-related genomics and transcriptomics publications). The genomic sequence and RNA-Seq data will comprise of the main contents of this database. Detailed information regarding samples, data quality and other information regarding these data will also be provided to researchers to facilitate further analyses. As a discovery tool, this database and analytical platform will help researchers to identify biological mechanisms in BR and design further experiments using its main modules for comparative genomics, protein-protein interaction, co-expression network and regulated network analyses.

Conclusions

The GABR project is the largest international, collaborative scientific project for the study of BR to date. As the world's first large-scale multi-omics project for BR, the GABR project will not only greatly facilitate conservation of global BR biodiversity and sustainable utilization of natural BR resources, but will also provide valuable data for boosting BR research and expanding the understanding of BR genetics and other biology. With more than 300 species of bamboo and two species of rattan to be sequenced, the first phase of GABR will soon be finished and will provide the first comprehensive dataset for BR. These data will begin to shed light on the mechanisms of important biological processes in BR.

Additional files

Additional file 1. Bamboo production and utilization.

Additional file 2. Summary of BR transcriptome data in Sequence Read Archive (SRA) at NCBI.

Additional file 3. List of the current GABR Project Consortium members.

Additional file 4. Location information on bamboo and rattan in China.

Additional file 5. Genome survey results of two rattan species (*Daemonorops jenkinsiana* and *Calamus simplicifolius*).

Abbreviations

The GABR Project: the Genome Atlas of Bamboo and Rattan Project; Bamboo-T1K: Transcriptomes of 1,000 bamboos; Rattan-G5: Genomes of 5 rattans; BR: bamboo and rattan; ICBR: International Centre for Bamboo and Rattan; INBAR: International Network for Bamboo and Rattan; RNA-Seq: RNA sequencing; NCBI: National Center for Biotechnology Information; SRA: Sequence Read Archive; SOPs: standard operating procedures.

Competing interests

The authors declare that they have no competing interests.

Authors' contributions

HSZ, ZMG and XL drafted the original text with detailed input from other authors and all authors have read and approved the final manuscript and participated in the GABR project.

Acknowledgements

We wish to acknowledge the GABR Consortium members, partners, advisors and supporters who have helped the GABR project run smoothly.

Funding

The work was supported by the Sub-Project of the National Science and Technology Support Plan of the Twelfth Five-Year Plan in China [No. 2015BAD04B03 and No. 2015BAD04B01], the National Science Foundation of China [No. 31400557], and Fundamental Research Funds for the International Center for Bamboo and Rattan [No. 1632016013]

Reference

1. Jiang Z: **Bamboo and Rattan in the World**. *China Forestry Publishing House* 2007, **Beijing, China**.
2. International Network of Bamboo and Rattan (INBAR): **International trade of bamboo and rattan products (Chinese)**. INBAR trade report, 2013.
3. Peng Z, Lu Y, Li L, Zhao Q, Feng Q, Gao Z, Lu H, Hu T, Yao N, Liu K *et al*: **The draft genome of the fast-growing non-timber forest species moso bamboo (*Phyllostachys heterocycla*)**. *Nat Genet* 2013, **45**(4):456-461, 461e451-452.
4. The Wellcome Trust (Fort Lauderdale January 14–15: **Sharing data from large-scale biological research projects: a system of tripartite responsibility.** 2003.

5. Toronto International Data Release Workshop A, Birney E, Hudson TJ, Green ED, Gunter C, Eddy S, Rogers J, Harris JR, Ehrlich SD, Apweiler R *et al*: **Prepublication data sharing**. *Nature* 2009, **461**(7261):168-170.

6. Zhao H, Peng Z, Fei B, Li L, Hu T, Gao Z, Jiang Z: **BambooGDB: a bamboo genome database with functional annotation and an analysis platform**. *Database : the journal of biological databases and curation* 2014, **2014**:bau006.

Figure legend

Figure 1. Example of phenotypical diversity in bamboo. Shoots of different bamboo species are shown to reflect phenotypical diversity in bamboo (From 1 to 9, the species are *Oligostachyum sulcatum, Phyllostachys atrovaginata, Phyllostachys aurea, Phyllostachys elegans, Phyllostachys nigra var. henonis, Phyllostachys incarnata, Phyllostachys nidularia, Phyllostachys flexuosa, Phyllostachys parvifolia*).

Figure 2. Global map of the distributions of bamboo and rattan within existing forests. According to UNEP-WCMC-INBAR (the United Nations Environment Programme's World Conservation Monitoring Centre and International Network for Bamboo and Rattan) Reports in 2003 and 2004 and INBAR research on bamboo and rattan distribution, bamboos (a) are distributed in 87 countries or regions, and rattans (b) are distributed in 35 countries or regions.

Tables

Table 1. List of bamboo and rattan genera and species included in the GABR project

Genera*	Species Number		
	Total	DNA Barcode	RNA-Seq
Acidosasa	6	5	5
Ampelocalamus	2	1	1
Bambusa	100	54	30
Bashania	4	2	2
Brachystachyum	1	2	1
Cephalostachyum	20	10	8
Chimonobambusa	20	5	5
Chimonocalamus	10	1	1
Dendrocalamopsis	9	7	7
Dendrocalamus	40	16	12
Drepanostachyum	10	3	3
Fargesia	80	15	5
Ferrocalamus	1	1	1
Gelidocalamus	9	2	2
Gigantochloa	30	6	3
Indocalamus	20	10	10
Indosasa	15	7	6
Melocalamus	3	2	2
Melocanna	2	1	1
Metasasa	2	1	1

Monocladus	3	1	1
Neomicrocalamus	2	1	1
Neosinocalamus	2	6	2
Oligostachyum	15	5	5
Phyllostachys	50	95[#]	47
Pleioblastus	50	19	10
Pseudosasa	30	11	8
Pseudostachyum	1	1	1
Qiongzhuea	8	2	2
Sasa	37	6	5
Schizostachyum	50	5	5
Semiarundinaria	10	2	2
Shibataea	7	4	4
Sinobambusa	13	8	6
Thamnocalamus	2	1	1
Thyrsostachys	2	1	1
Yushania	60	20	10
Total	**726**	**339**	**217**

[*] Genera mainly distributed in Asia, detailed information from Flora of China
[#] Including some variants

Table 2. Topics of ongoing subprojects in the GABR project

No.	'Omics Data types				Topics of subprojects
	G	T	P	M	
1					Bambusoideae evaluation based on nuclear phylogenomics
2					Identification of bamboo species using DNA barcodes
3					Genome sequencing and assembly for the rattans
4					Cellular and molecular characterization of single internode growth of bamboo
5					Transcriptome analysis to reveal the mechanism controlling shortened internodes in bamboo
6					Genome-wide profiling of non-coding circular RNAs in bamboo
7					Comprehensive analysis of seasonal phytochemical changes in bamboo as food for captive giant panda
8					Integrated transcriptomics and metabolomics approaches to reveal terpenoid biosynthesis pathways in bamboo.
9					Floral transcriptome reveals surprising protein sequence conservation in the flowering pathway
10					Gene network analysis and functional module identification for bamboo
11					A pipeline for plant genome annotation developed for high-throughput sequence data of bamboo and rattan

G: Genome; T: Transcriptome; P: Proteome; M: Metabolome

Click here to download Figure Figure 1.pdf

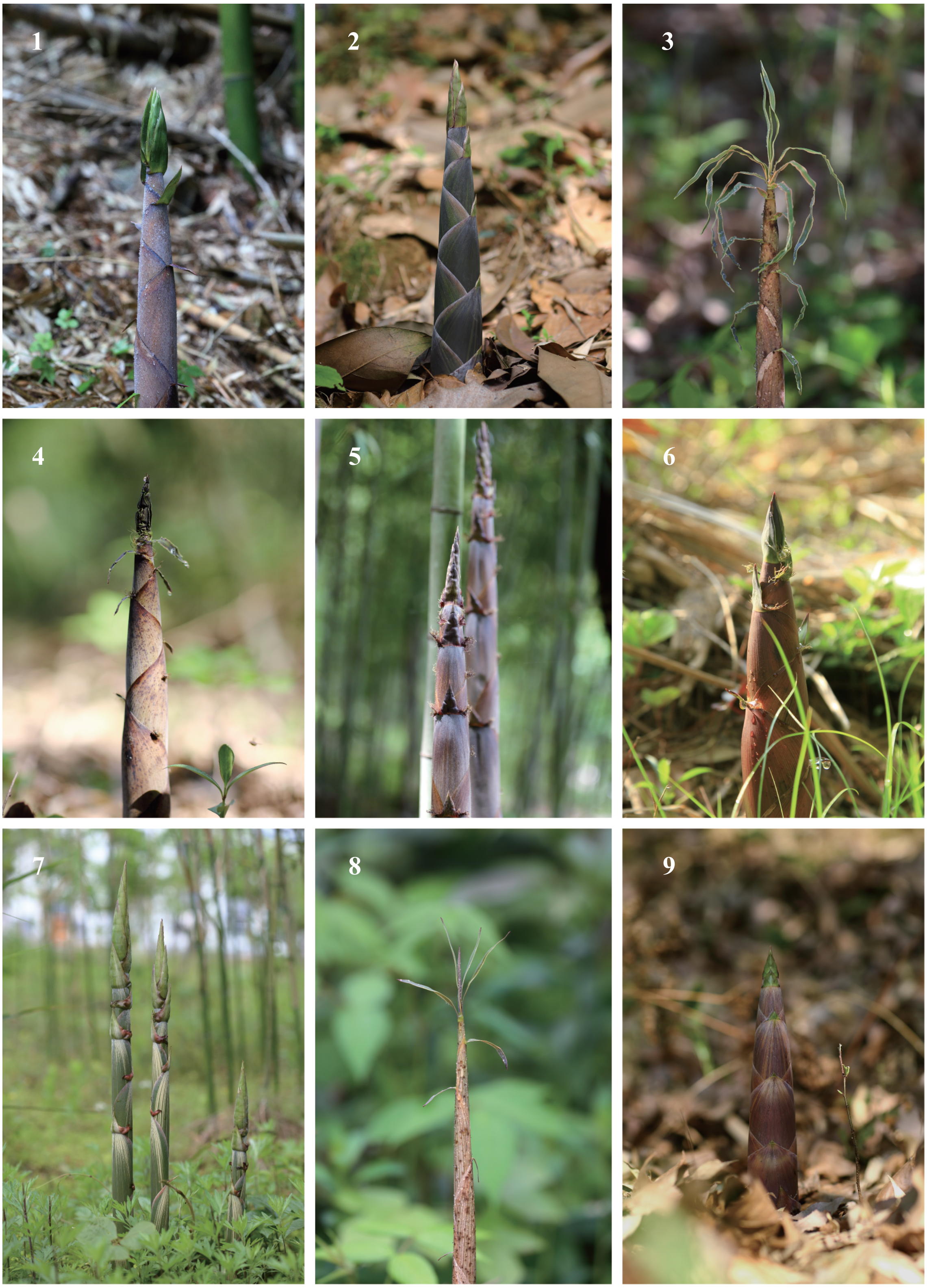

Figure 2

Click here to download Figure Figure 2.pdf

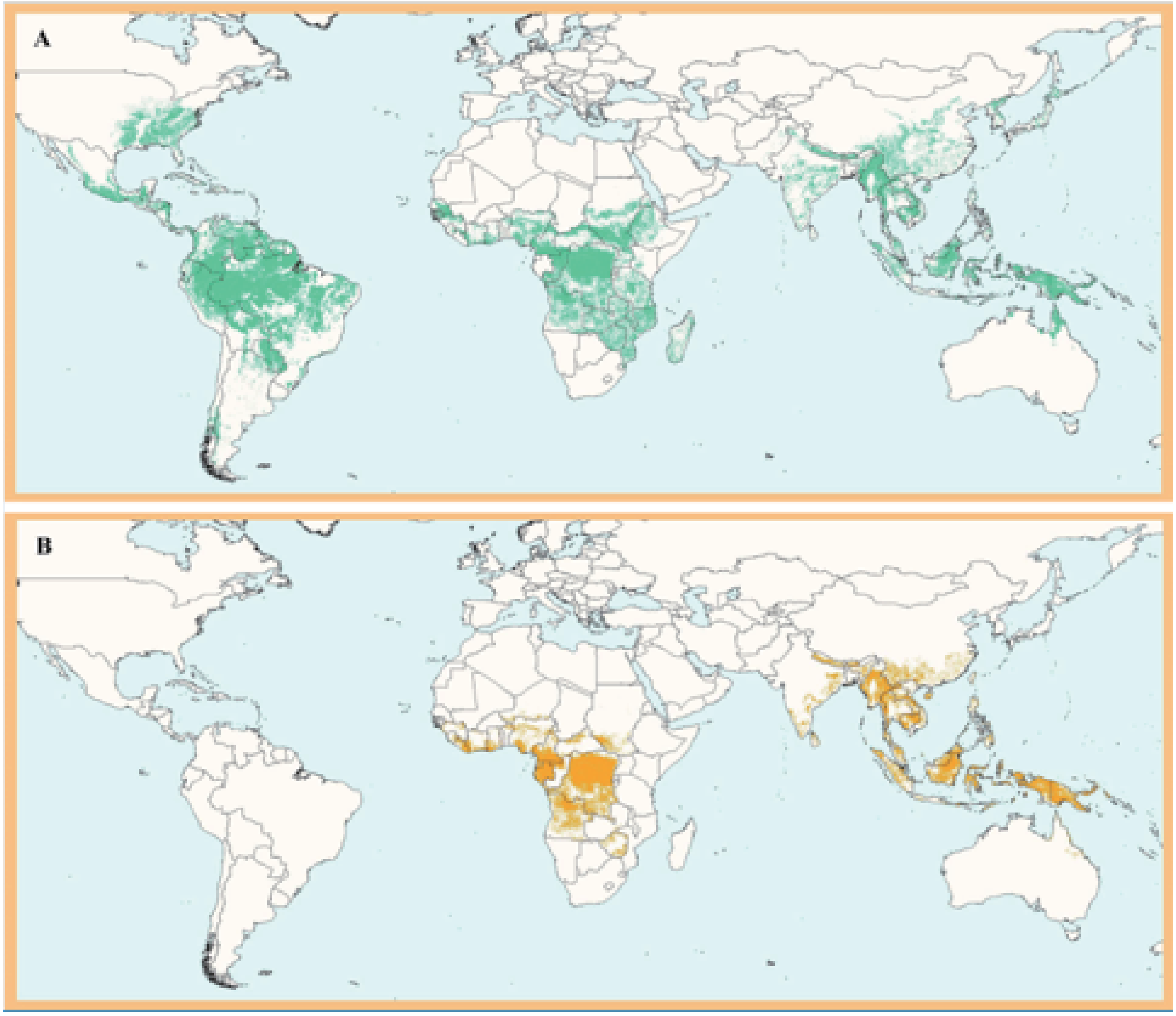

Additional file 1

Click here to access/download
Supplementary Material
Additional file 1.xlsx

Additional file 2

Click here to access/download
Supplementary Material
Additional file 2.xlsx

Additional file 3

Click here to access/download
Supplementary Material
Additional file 3.xlsx

Additional file 4

Click here to access/download
Supplementary Material
Additional file 4.xlsx

Additional file 5

Click here to access/download
Supplementary Material
Additional file 5.xlsx
