# Supplementary material for: Announcing the Genome Atlas of Bamboo and Rattan (GABR) project: promoting research in evolution and in economically and ecologically beneficial plants
Source: Gigascience. 2017 Jun 16;6(7):1–7. doi: 10.1093/gigascience/gix046 (PMC5570132; doi:10.1093/gigascience/gix046)
Supplement: GIGA-D-17-00120_Revision-1.pdf [file gix046_GIGA-D-17-00120_Revision-1.pdf]

Manuscript Click here to download Manuscript commentary.docx

Commentary

Announcing the Genome Atlas of Bamboo and Rattan (GABR) project: promoting research in evolution and in economically and ecologically beneficial plants

Hansheng Zhao[1,‡], Shancen Zhao[2,‡], International Network for Bamboo and Rattan[3,‡], Benhua Fei[1], Huan Liu[2], Huanming Yang[2], Honghai Dai[1], Dan Wang[1], Wei Jin[3], Feng Tang[1], Qiang Gao[2], Hang Xun[1], Yuwei Wang[1], Lianghua Qi[1,4,5], Xianghua Yue[1,6], Shuyan Lin[7], Lianfeng Gu[8], Lubin Li[9], Tiansheng Zhu[10], Qiang Wei[11,12], Zhen Su[13], Tarmeze Bin Wan Ariffin Wan[14], Daniel A. Ofori[15], George Mbeva Muthike[16], Yigardu Mulatu Mengesha[17], Roberto Magno de Castro e Silva[18], Antonio Ludovico Beraldo[19], Zhimin Gao[1,*], Xin Liu[2,*], and Zehui Jiang[1,*]

[*]Correspondence addresses:

ZG: State Forestry Administration Key Open Laboratory on the Science and Technology of Bamboo and Rattan, International Center for Bamboo and Rattan, Beijing 100102, China; Tel: +86-010-84789803; E-mail: gaozhimin@icbr.ac.cn

XL: BGI-Shenzhen, Shenzhen 518083, China; Tel: +86-755-36307888; E-mail: liuxin@genomics.cn

ZJ: State Forestry Administration Key Open Laboratory on the Science and Technology of Bamboo and Rattan, International Center for Bamboo and Rattan, Beijing 100102, China; Tel: 010-84789803; E-mail: jiangzehui@icbr.ac.cn

‡ Equal contributors

[1]State Forestry Administration Key Open Laboratory on the Science and Technology of Bamboo and Rattan, International Center for Bamboo and Rattan, Futongdong Rd, WangJing, Chaoyang District 100102, Beijing, China;

[2]BGI-Shenzhen, Main Building Beishan Industrial Zone, Yantian District, Shenzhen 518083, Guangdong, China;

[3]International Network for Bamboo and Rattan, Futongdong Rd, Wangjing, Chaoyang District 100102, Beijing, China;

[4]Hainan Sanya National Positioning and Monitoring Station for Ecosystem of Bamboo and Rattan Associated Forest, Xincheng Rd, Tianya District, Sanya 572000, Hainan, China;

[5]Research Institute of Tropical Forest Plants, International Center for Bamboo and Rattan, Xincheng Rd, Tianya District, Sanya 572000, Hainan, China;

[6]Anhui Taiping Experimental Station, International Center for Bamboo and Rattan, Cuiwei Rd, Huangshan District, Huangshan 245716, Anhui, China;

[7]Nanjing Forestry University, Bamboo Research Institute, Longpan Rd, Xuanwu District, Nanjing 210037, Jiangsu, China;

[8]Basic Forestry and Proteomics Research Center, Haixia Institute of Science and Technology, Fujian Agriculture and Forestry University, Shangxiadian Rd, Cangshan District, Fuzhou 350002, Fujian, China;

[9]Research Institute of Forestry, Chinese Academy of Forestry, Xiangshan Rd, Haidian District 100091, Beijing, China;

[10]School of Computer Science, Fudan University, Handan Rd, Yangpu District 200433, Shanghai, China;

[11]Co-Innovation Center for Sustainable Forestry in Southern China, Nanjing Forestry University, Longpan Rd, Xuanwu District, Nanjing 210037, Jiangsu, China;

[12]Bamboo Research Institute, Nanjing Forestry University, Longpan Rd, Xuanwu District, Nanjing 210037, Jiangsu, China;

[13]State Key Laboratory of Plant Physiology and Biochemistry, College of Biological Sciences, China Agricultural University, Yuanmingyuan W Rd, Haidian District 100193, Beijing, China;

[14]Forest Products Division, Forest Research Institute Malaysia, Jalan Frim, Kepong, Kuala Lumpur 52109, Selangor, Malaysia

[15]Forestry Research Institute of Ghana, KNUST, Kumasi PO Box UP 63, Ashanti, Ghana;

[16]Kenya Forestry Research Institute, Muguga off Nairobi-Nakuru Highway, 20412-00200, Nairobi, Kenya;

[17]Ethiopian Environment and Forest Research Institute, Gurd Shola, 24536-1000, Addis Ababa, Ethiopia;

[18]Federal University of Goiás, R. Riachuelo, Setor Samuel Graham, Jataí - GO, 75804-020, Goiás, Brazil;

[19]School of Agricultural Engineering, University of Campinas, Av. Cândido Rondon, 501 - Cidade Universitária, Campinas - SP, 13083-875, São Paulo, Brazil

E-mail addresses:

Hansheng Zhao: zhaohansheng@icbr.ac.cn

Shancen Zhao: zhaoshancen@genomics.cn

International Network for Bamboo and Rattan: zyli@inbar.int

Benhua Fei: feibenhua@icbr.ac.cn

Huan Liu: liuhuan@genomics.cn

Huanming Yang: yanghuanming@genomics.cn

Honghai Dai: h_hdai@icbr.ac.cn

Dan Wang: wangdan@icbr.ac.cn

Wei Jin: wjin@inbar.int

Feng Tang: tangfeng@icbr.ac.cn

Qiang Gao: gaoqiang@genomics.cn

Hang Xun: xunhang@icbr.ac.cn

Yuwei Wang: wangyuwei@icbr.ac.cn

Lianghua Qi: qlh@icbr.ac.cn

Xianghua Yue: yuexianghua@icbr.ac.cn

Shuyan Lin: 690952866@qq.com

Lianfeng Gu: lfgu@fafu.edu.cn

Lubin Li: lilubin@126.com

Tiansheng Zhu: tszhu@fudan.edu.cn

Qiang Wei: weiqiang@njfu.edu.cn

Zhen Su: zhensu@cau.edu.cn

Tarmeze Bin Wan Ariffin Wan: drwantarmeze@gmail.com

Daniel A. Ofori: dodori12@gmail.com

George Mbeva Muthike: muthikegm@yahoo.com

Yigardu Mulatu Mengesha: yigardumulatu@gmail.com

Roberto Magno de Castro e Silva: roberto@embambu.com.br

Antonio Ludovico Beraldo: beraldo@gmail.com

Zhimin Gao: gaozhimin@icbr.ac.cn

Xin Liu: liuxin@genomics.cn

Zehui Jiang: jiangzehui@icbr.ac.cn

Abstract

Bamboo and rattan are widely grown for manufacturing, horticulture, and agroforestry. Bamboo and rattan production might help reduce poverty, boost economic growth, mitigate climate change, and protect the natural environment. Despite progress in research, sufficient molecular and genomic resources to study these species are lacking. We launched the Genome Atlas of Bamboo and Rattan (GABR) project, a comprehensive, coordinated international effort to accelerate understanding of BR genetics through genome analysis. GABR includes two core subprojects: Bamboo-T1K (Transcriptomes of 1000 Bamboos) and Rattan-G5 (Genomes of 5 Rattans), and several other subprojects. Here we describe the organization, directions and status of GABR.

Keywords: GABR; bamboo; rattan; large-scale; multi-omics; biodiversity

Introduction

Bamboo species belong to the grass family Poaceae, subfamily Bambusoideae, and exhibit substantial phenotypic diversity (Figure 1 and Figure 2). Approximately 1250 bamboo species have

been reported across 75 genera, occupying a range of environments around the world, from tropical and warm temperate ecosystems to cold temperate regions [1] (Figure 3A).

Rattans are spiny climbing palms in the Arecaceae family, subfamily Calamoideae. Native to tropical and subtropical regions in the Eastern Hemisphere, more than half of the ~600 rattan species across 13 genera are distributed in Asia. The remaining species are found in West Africa and on islands in the northern Pacific [1] (Figure 3B).

Bamboos and rattans (BR) are produced for food and energy, but their special fiber and wood characteristics mean they are also used industrially on a large scale for fiber, building materials, and utensils. Growing BR has great potential for poverty reduction, industrial development and sustainable development.

Plant-derived natural resources are threatened by environmental change. Unlike trees, which have long growth cycles, bamboo can be harvested every 3–5 years, and every 5–7 years for rattan, without causing deforestation or resource loss. Once their root systems are established underground, BR species can grow new shoots each year. The International Network for Bamboo and Rattan (INBAR) estimated that the global BR trade was worth ~$60 billion in 2015, and has increased by ~$2.5 billion annually [2]. Approximately 1.5 billion people worldwide are associated with the use or production of BR resources [2].

Molecular genetic research is important to promote genetic, evolutionary, taxonomic, and functional BR studies to comprehensively understand the biology and other characteristics of these genera, and to rationally utilize BR resources. Globally, the International Center for Bamboo and Rattan plays a significant role in BR research and development. It participated in the Bamboo Genome Project,

and in 2013 released the draft genome of Moso bamboo (*Phyllostachys edulis*) [3], the only species for which a whole genome sequence is publicly available. Other genomic BR resources remain limited: 53 transcriptomes from two bamboo species, and 8 transcriptomes of a single rattan species are publicly available in the National Center for Biotechnology Information (NCBI) Sequence Read Archive (SRA) database [4] (Additional file 1).

The lack of extensive genomic resources seriously impedes progress in BR classification, and evolutionary and functional BR analyses. Therefore, we launched the Genome Atlas of Bamboo and Rattan (GABR) project, which aims to generate large-scale 'omics data to advance BR studies ranging from basic molecular biology research to applied genetic engineering.

The GABR Consortium

The GABR Consortium was established in 2016 by INBAR, an intergovernmental organization founded in 1997 through a United Nations treaty. Members of INBAR come from the many countries with major bamboo and rattan resources. It has a professional team comprising experts in bamboo and rattan, forestry, natural resource management, ecosystem services, socioeconomics, capacity building, and knowledge sharing. The GABR Consortium, which is headquartered within the International Center for Bamboo and Rattan in Beijing, was initiated as an international, collaborative, non-profit initiative to generate BR genome sequences and other 'omics datasets to help improve the conservation and utilization of the world's BR resources. The consortium now consists of ~100 scientists from 52 scientific institutions and universities across 42 countries, and one intergovernmental organization (Additional file 2). Two core consortium members, who are

recognized experts in taxonomy, bioinformatics, phylogeny and evolution, form the Steering Committee. BGI (formerly known as Beijing Genomics Institute) in Shenzhen, China, was chosen to facilitate sample collection and data sharing. INBAR provides support for the GABR project.

The GABR Consortium invites international experts and institutions from related fields to participate in the project. Interested participants should i) provide BR resources not included in the current sample list (Table 1); ii) contribute to data generation; or iii) use GABR ‘omics data to address BR research questions. Brief proposals should be e-mailed to Professor Zhimin Gao at GABR@icbr.ac.cn, stating intended contributions to the GABR project, and a detailed research plan. Proposals will be reviewed, and if appropriate, applicants will be accepted as new project members. Otherwise, reasons for rejection and suggestions on how to improve applications will be provided.

Project development and current progress

The GABR project includes two core subprojects: Bamboo-T1K (Transcriptomes of 1000 Bamboos) and Rattan-G5 (Genomes of 5 Rattans). Several critical BR studies using high-throughput sequencing data were also included.

The first phase of the GABR project has three main components: 1) nuclear phylogenomic analyses to reveal the phylogenetic relationships between bamboo genera, using data from ~220 representative Old World woody bamboo species from 37 genera (item 1 in Table 2), and classification studies of ~340 bamboo genera using transcriptomic sequencing and DNA barcode analysis technologies (item 2); 2) whole-genome sequencing and genome assembly of two rattan species (*Daemonorops jenkinsiana* and *Calamus simplicifolius*) of economic significance (item 3);

and 3) use of large-scale multi-omics data to explore critical biological bamboo phenotypes, including the bamboo rapid-growth trait (items 4–6), flower development (item 7), and the regulation of important metabolites using proteomics and metabolomics technologies (items 8 and 9). Large-scale multi-omics data will also be systematically and comprehensively analyzed using in-depth data-mining methods (Item 10). For this work, we will develop novel bioinformatics methods or pipelines for assembling and annotating large genomes and for multi-omics analyses in plants (item 11).

Sampling and data generation has been initiated. GABR has established a collaborative global network to collect ~340 bamboo and two rattan samples from Malaysia, Ghana, Kenya, Ethiopia, Brazil, and many locations in China. For the two rattan samples, flow cytometry analysis and a whole genome survey have been conducted to estimate the genome size (unpublished work). Project members are currently carrying out DNA and RNA extraction on other samples, as well as data generation.

GABR data sharing policies

Following the Bermuda and Fort Lauderdale agreements [5], and the Toronto International Data Release Workshop guidelines [6], data will be shared in a timely manner, and ahead of any publication of results, at the official GABR website [7] (formerly known as the Bamboo Genome Database [BambooGDB]) [8]. Raw sequence data generated by the GABR project and passing quality evaluation criteria will also be deposited in the NCBI SRA [4].

To facilitate our understanding of BR genomes and future studies related to the GABR project, we will also develop a GABR website where all available public BR data will be aggregated, whether from the GABR project or from previous BR-related genomics and transcriptomics publications. This database will mainly comprise genomic sequences and RNA sequencing data. Detailed information regarding samples, data quality and other information will also be provided to researchers to facilitate further analyses. As a discovery tool, this database and analytical platform will help researchers to identify biological BR mechanisms, and to design further experiments using its modules for comparative genomics, protein–protein interactions, co-expression networks and regulated network analyses.

Conclusions

As the largest international, collaborative scientific project for the study of BR to date, and the world's first large-scale multi-omics project for BR, GABR will help to conserve global BR biodiversity and sustainable use of natural BR resources. It will also provide valuable data to boost BR research and expand our understanding of BR genetics and biology. More than 300 species of bamboo and two species of rattan will be sequenced. The first phase of GABR is almost complete, and will provide the first comprehensive dataset for BR resources. These data will shed light on the mechanisms of important biological BR processes.

Additional Files

Additional file 1. Summary of BR transcriptome data in Sequence Read Archive (SRA) at NCBI.

Additional file 2. List of the current GABR Project Consortium members.

.

Abbreviations

Bamboo-T1K, Transcriptomes of 1000 Bamboos; BR: bamboo and rattan; GABR Project, Genome Atlas of Bamboo and Rattan Project; INBAR, International Network for Bamboo and Rattan; NCBI: National Center for Biotechnology Information; Rattan-G5, Genomes of 5 Rattans; SRA, Sequence Read Archive.

Acknowledgements

We wish to acknowledge the GABR Consortium members (listed in Additional file 1), partners, advisors and supporters who have helped the GABR project run smoothly.

Funding

This work was supported by the Sub-Project of the National Science and Technology Support Plan of the Twelfth Five-Year Plan in China (grant numbers 2015BAD04B03 and 2015BAD04B01), the National Science Foundation of China (grant number 31400557), and by Fundamental Research Funds for the International Center for Bamboo and Rattan (grant number 1632016013).

Competing interests

The authors declare that they have no competing interests.

Authors' contributions

H.S.Z., Z.M.G. and X.L. drafted the original manuscript text with detailed input from other authors. All authors participated in the GABR project, and have read and approved the final manuscript.

References

1. Jiang Z. Bamboo and Rattan in the World. Beijing: China Forestry Publishing House, 2007,.
2. International Network of Bamboo and Rattan Trade Report. International trade of bamboo and rattan products [Chinese]. Beijing: International Network of Bamboo and Rattan, 2013.
3. Peng Z, Lu Y, Li L et al. The draft genome of the fast-growing non-timber forest species moso bamboo (*Phyllostachys heterocycla*). Nat Genet 2013;45(4):456–61, 461e1–2.
4. National Center for Biotechnology Information Sequence Read Archive. https://www.ncbi.nlm.nih.gov/sra. Accessed 20 May 2017.
5. Wellcome Trust. Sharing data from large-scale biological research projects: a system of tripartite responsibility. Report of a meeting organized by the Wellcome Trust and held on 14–15 January 2003 at Fort Lauderdale, USA. London: Wellcome Trust, 2003.
6. Toronto International Data Release Workshop Authors, Birney E, Hudson TJ et al. Prepublication data sharing. Nature 2009;461(7261):168–70.
7. The GABR Project. http://www.gabr-project.com/. Accessed 10 June 2017.

8. Zhao H, Peng Z, Fei B et al. BambooGDB: a bamboo genome database with functional annotation and an analysis platform. Database (Oxford) 2014;2014:bau006.

9. 'eFloras (2008). Published on the Internet http://www.efloras.org [Accessed 20 May 2017]' Missouri Botanical Garden, St. Louis, MO & Harvard University Herbaria, Cambridge, MA.

Figure legends

Figure 1. Phenotypic diversity in bamboo shoot. Shoots of different bamboo species are shown to reflect phenotypic diversity in bamboo shoot. 1, *Oligostachyum sulcatum*; 2, *Phyllostachys atrovaginata*; 3, *P. aurea*; 4, *P. elegans*; 5, *P. nigra* var. *henonis*; 6, *P. incarnate*; 7, *P. nidularia*; 8, *P. flexuosa*; 9, *P. parvifolia*.

Figure 2. Phenotypic diversity in bamboo culm. Culms of different bamboo species are shown to reflect phenotypic diversity in bamboo culm. 1, *Phyllostachys edulis* f. *tubaeformis*; 2, *Phyllostachys edulis* ‘Kikko-chiku’; 3, *Bambusa ventricosa*; 4, *Phyllostachys edulis* f. *holochrysa*; 5, *Phyllostachys edulis* f. *luteosulcata*; 6, *Phyllostachys violascens* f. *viridisulcata*; 7, *Phyllostachys nigra*; 8, *Phyllostachys bambusoides* f. *lacrima-deae*; 9, *Bambusa multiplex* ‘Alphonse-Karr’.

Figure 3. Global map of the distribution of bamboo and rattan in existing forests. According to the United Nations Environment Program’s World Conservation Monitoring Centre and International Network for Bamboo and Rattan Reports in 2003 and 2004, and research by the International Network of Bamboo and Rattan on bamboo and rattan distribution, bamboo species (A) are found in 87 countries or regions, and rattan species (B) are found in 35 countries or regions.

Tables

Table 1. List of bamboo and rattan genera and species included in the GABR project

Genera[1]	Number of species[2]		
	Number of species included in the GABR project	Number of species to generate DNA barcodes	Number of species to generate RNA sequencing data
Acidosasa	6	5	5
Ampelocalamus	2	1	1
Bambusa	100	54	30
Bashania	4	2	2
Brachystachyum	1	2	1
Cephalostachyum	20	10	8
Chimonobambusa	20	5	5
Chimonocalamus	10	1	1
Dendrocalamopsis	9	7	7
Dendrocalamus	40	16	12
Drepanostachyum	10	3	3
Fargesia	80	15	5
Ferrocalamus	1	1	1
Gelidocalamus	9	2	2
Gigantochloa	30	6	3
Indocalamus	20	10	10
Indosasa	15	7	6
Melocalamus	3	2	2

Melocanna	2	1	1
Metasasa	2	1	1
Monocladus	3	1	1
Neomicrocalamus	2	1	1
Neosinocalamus	2	6	2
Oligostachyum	15	5	5
Phyllostachys	50	95[#]	47
Pleioblastus	50	19	10
Pseudosasa	30	11	8
Pseudostachyum	1	1	1
Qiongzhuea	8	2	2
Sasa	37	6	5
Schizostachyum	50	5	5
Semiarundinaria	10	2	2
Shibataea	7	4	4
Sinobambusa	13	8	6
Thamnocalamus	2	1	1
Thyrsostachys	2	1	1
Yushania	60	20	10
Total	**726**	**339**	**217**

1. Genera mainly distributed in Asia. Detailed information about each genus is available from Flora of China [9].
2. We listed the number of species to be studied in GABR (number of species included in the GABR project), the number of species to generate DNA barcodes in GABR (number of species to generate DNA barcodes) and the number of species to generate RNA sequencing data in GABR (number of species to generate RNA sequencing data).

Table 2. Topics of ongoing subprojects in the GABR project

Item No.	Data types[1] G	T	P	M	Subproject topics
1	√	√			Bambusoideae evaluation based on nuclear phylogenomics
2	√	√			Identification of bamboo species using DNA barcodes
3	√	√			Genome sequencing and assembly for rattan species
4	√	√			Cellular and molecular characterization of single internode growth of bamboo
5	√	√			Transcriptome analysis to reveal the mechanism controlling shortened internodes in bamboo
6	√	√			Genome-wide profiling of non-coding circular RNAs in bamboo
7	√	√	√	√	Comprehensive analysis of seasonal phytochemical changes in bamboo as food for captive giant panda
8	√	√	√	√	Integrated transcriptomics and metabolomics approaches to reveal terpenoid biosynthesis pathways in bamboo.
9	√	√	√		Transcriptome and proteome of bamboo related to floral developing
10	√	√	√	√	Gene network analysis and functional module identification for bamboo
11	√	√	√	√	A pipeline for plant genome annotation developed for high-throughput sequence data of bamboo and rattan

Click here to download Figure Figure 1.pdf

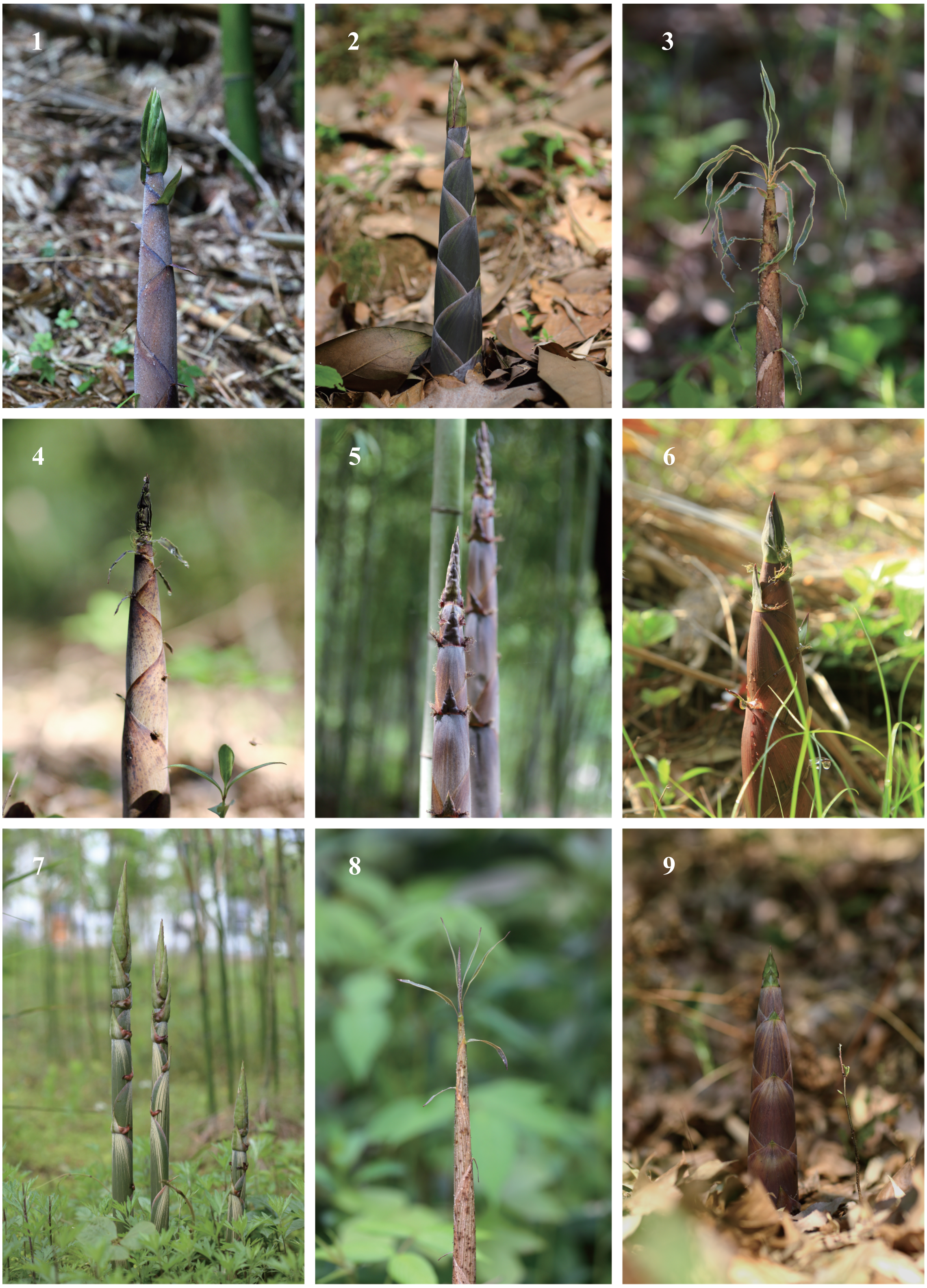

Figure 2

Click here to download Figure Figure 2.pdf

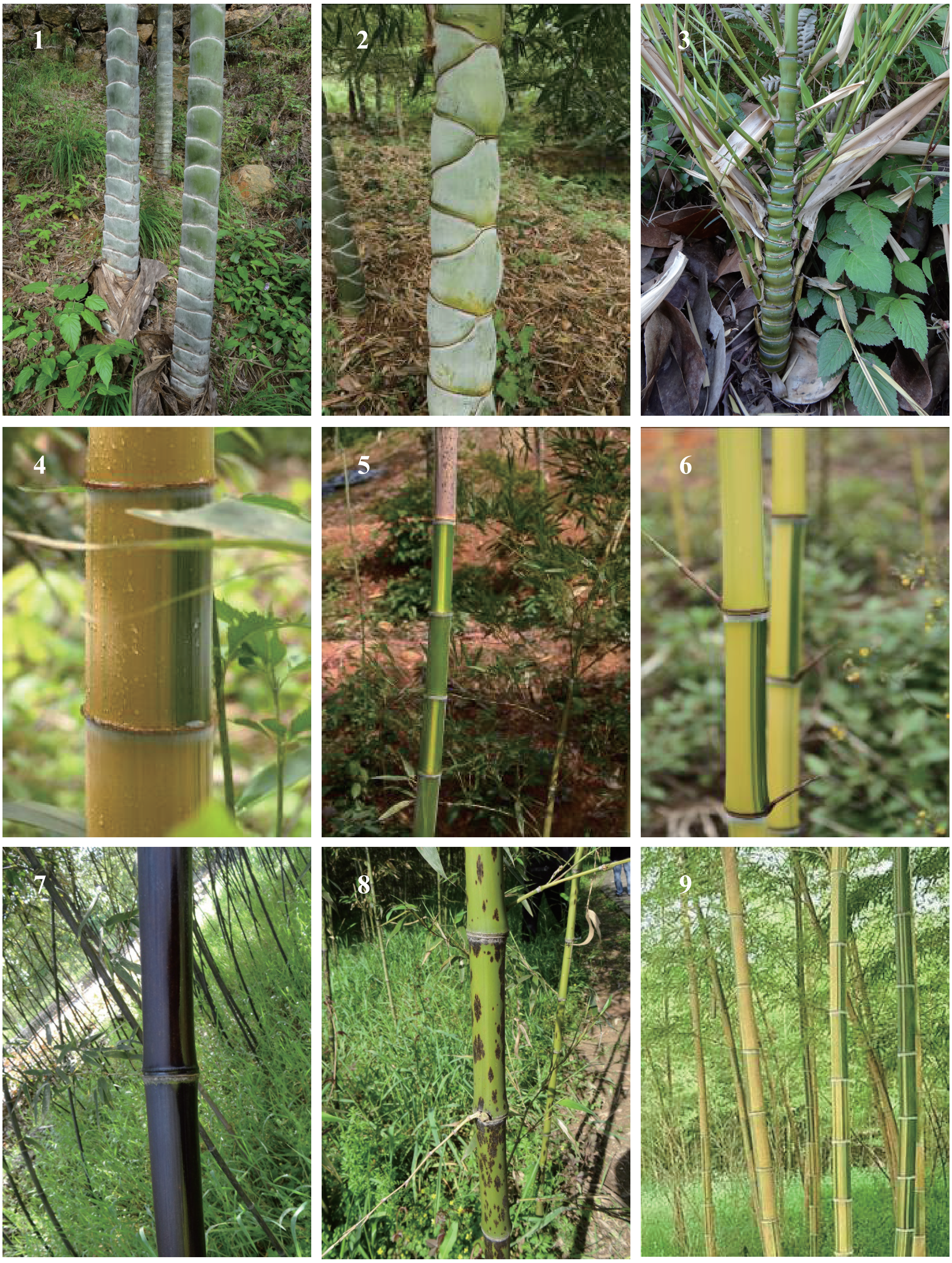

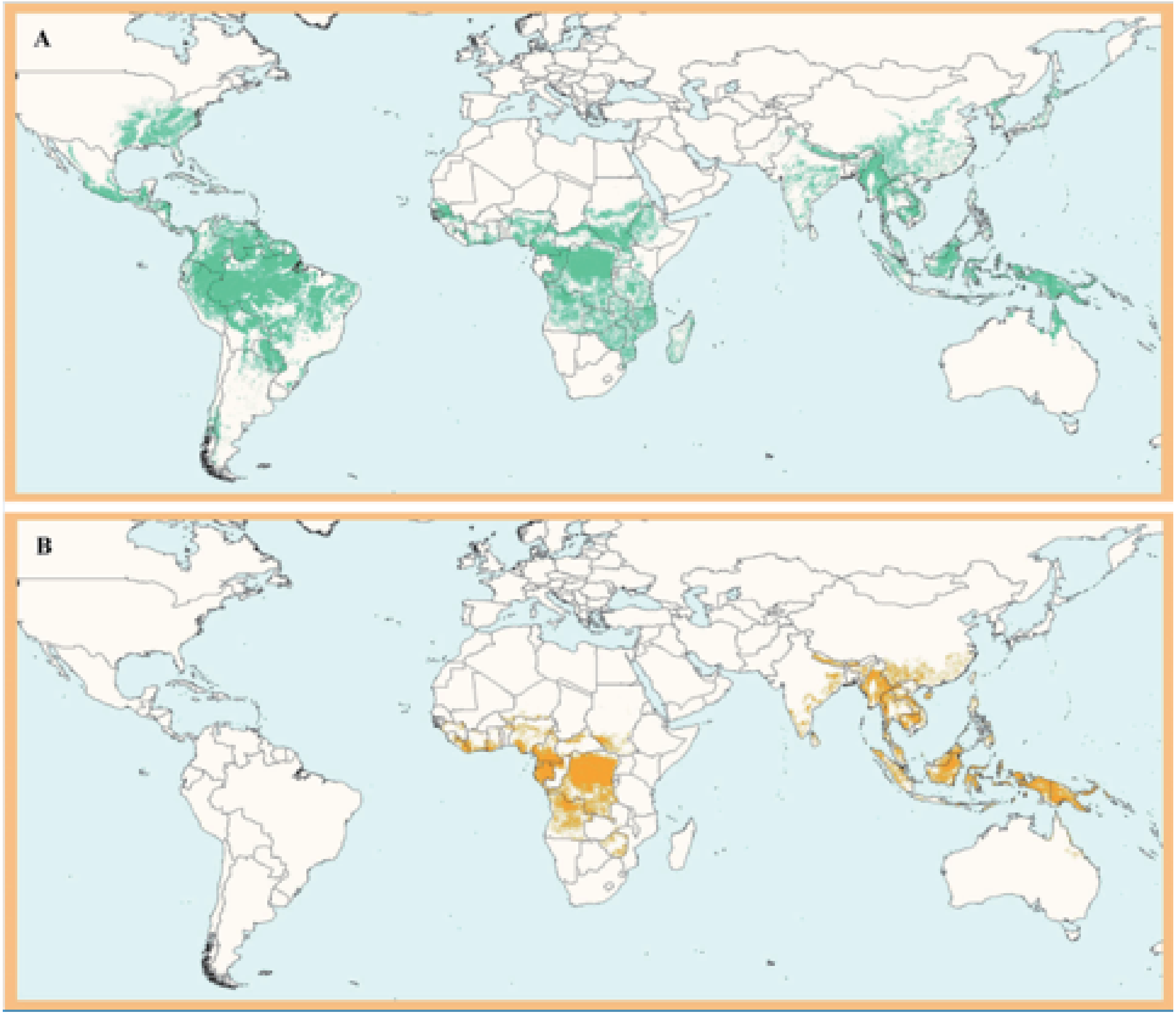
A
B

Click here to access/download
Supplementary Material
Additional file 1.xlsx

Click here to access/download
Supplementary Material
Additional file 2.xlsx
