# Supplementary material for: Announcing the Genome Atlas of Bamboo and Rattan (GABR) project: promoting research in evolution and in economically and ecologically beneficial plants
Source: Gigascience. 2017 Jun 16;6(7):1–7. doi: 10.1093/gigascience/gix046 (PMC5570132; doi:10.1093/gigascience/gix046)
Supplement: Response-to-Reviewer-Comments_Original-Submission.pdf [file gix046_Response-to-Reviewer-Comments_Original-Submission.pdf]

Author's Response To Reviewer Comments

We have modified the manuscript according to the comments and suggestions of the editor. The major revisions are:
1) we have deleted the additional files only to remain the original Additional file 2 (the current Additional file 1), and the original Additional file 3 (the current Additional file 2). Especially for the original Additional file 5, it is the unpublished data thus we have indicated it in the context instead of providing the file.

2) We have included a new figure (current Figure 2) to show the diversity of bamboo culms according to the editor's suggestion.

For some minor points raised by the editor, we have the following description in addition to acceptance of the revisions:
1) We have included the phone number of the corresponding authors. For the addresses, we have included the full address. And for the PO box mailing addresses, we have contacted the authors to confirm.
2) For both Figure 1 and Figure 2, we haven't included the scale bar as the figures are showing the overall phenotype diversity, for which we think the current form should be good enough.
3) For the references, we have included one more reference for the Flora of China. And for the Reference 2, it only has a Chinese version thus we did not change it.
4) For Table 1, we have revised to include description of different columns.
